# Transcriptional and genomic mayhem due to aging-induced nucleosome loss in budding yeast

**DOI:** 10.15698/mic2014.04.139

**Published:** 2014-04-01

**Authors:** Zheng Hu, Kaifu Chen, Wei Li, Jessica K. Tyler

**Affiliations:** 1 Department of Biochemistry and Molecular Biology, UT MD Anderson Cancer Center, 6767 Bertner Ave, Houston, TX 77030.; 2 Dan L Duncan Cancer Center and Department of Molecular and Cellular Biology, Baylor College of Medicine, Houston, TX 77030.

**Keywords:** DNA damage, translocation, transcription, histones, yeast, aging

## Abstract

All eukaryotic genomes are assembled into a nucleoprotein structure termed chromatin, which is comprised of regular arrays of nucleosomes. Each nucleosome consists of eight core histone protein molecules around which the DNA is wrapped 1.75 times. The ultimate consequence of packaging the genome into chromatin is that the DNA sequences are relatively inaccessible. This allows the cell to use a comprehensive toolbox of chromatin-altering machineries to reveal access to the DNA sequence at the right time and right place in order to allow genomic processes, such as DNA repair, transcription and replication, to occur in a tightly-regulated manner. In other words, chromatin provides the framework that allows the regulation of all genomic processes, because the machineries that mediate transcription, repair and DNA replication themselves are relatively non-sequence specific and if the genome were naked, they would presumably perform their tasks in a random and unregulated manner. We recently provided support for this prediction in Zheng *et al.*, [Genes and Development (2014) 28: 396-408] by investigating a physiologically relevant scenario in which we had found that cells lose half of the core histone proteins, that is, during the mitotic aging (also called replicative aging) of budding yeast. Using new spike-in normalization techniques, we found that the occupancy of nucleosomes at most DNA sequences is reduced by 50%, leading to transcriptional induction of every single gene. This loss of histones during aging was also accompanied by a significantly-increased frequency of genomic instability including DNA breaks, chromosomal translocations, retrotransposition, and transfer of mitochondrial DNA into the nuclear genome (Figure 1).

The core histone protein sequences are the most conserved of all proteins across eukaryotes, indicating that the histones play a critical function that cannot tolerate sequence changes. The critical function of chromatin is further underscored by the fact that the cell expends a huge amount of energy on unpackaging the DNA from nucleosomes and rapidly repackaging it back into nucleosomes during every molecular transaction with the genome. As such, we were surprised to find that the level of histone proteins progressively declined during replicative aging. The reason for this age-associated histone loss appears to be due to a defect in protein synthesis because the histone protein half-life was not reduced, and the transcript levels were actually increased. Most importantly, histone loss seems to be causally involved in aging, because lifespan was extended when we overexpressed the histones.

**Figure 1:**
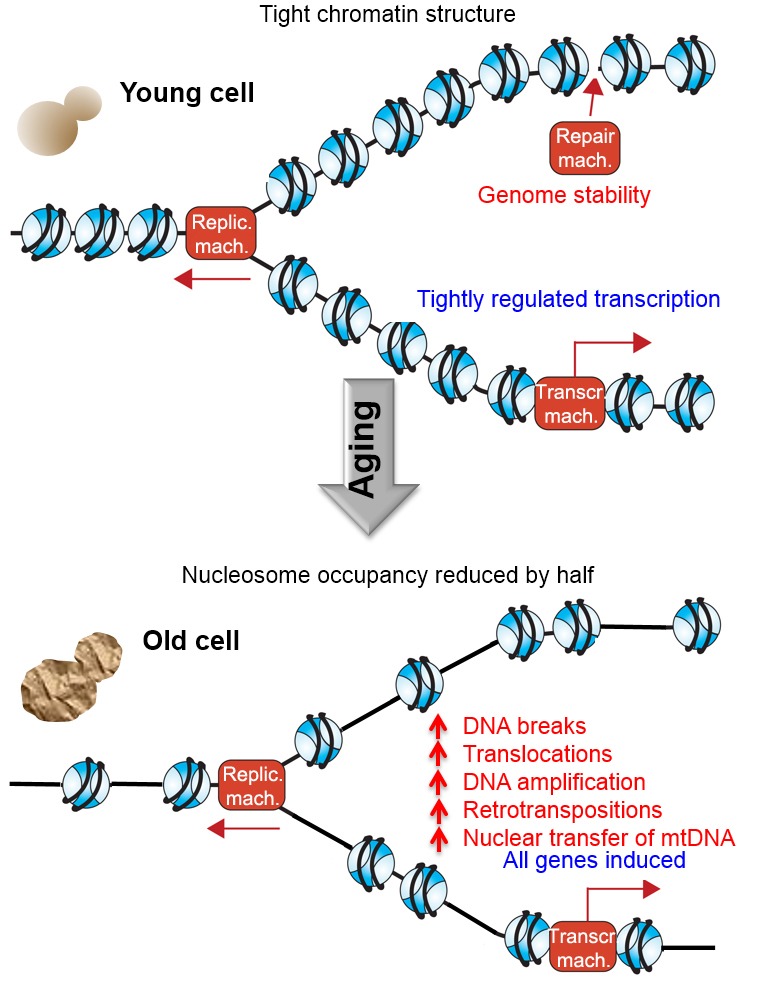
FIGURE 1: Summary of our findings. During replicative aging of budding yeast, there is a loss of half of the nucleosomes over the genome, leading to increased transcription from all genes in the genome, increased amounts of DNA breaks, translocations, amplifications, retrotransposition and transfer of mtDNA to the nucleus. Replic. mach., replication machinery; Repair mach., repair machinery; Transcr. mach., transcription machinery.

To confirm that the histones were indeed lost from the DNA we performed global nucleosome positioning analysis over the entire yeast genome in young and old cells, using high-throughput sequencing. In order to do this, we devised a spike-in control to allow normalization of the sequence read count relative to cell number, which had not previously been included in any published nucleosome mapping analyses. In this analysis we were asking: on what DNA sequences do nucleosomes chose to assemble in a cell when there is a limited number of histones? This was a relevant question because nucleosome occupancy regulates the functional activity of the underlying DNA sequence. Furthermore, others have shown that *in vitro*, the affinity of histone octamers for a given 147bp sequence varies over more than three orders of magnitude depending on the DNA sequence, suggesting that DNA sequence would be a major factor in determining where nucleosomes would be positioned on the DNA in a cell. However, this was not the case. In fact we found that nucleosomes in old cells occupy almost all the same DNA positions as they occupy in young cells (Fig. 2), albeit with 50% lower occupancy. Moreover, we found that the positioning of the nucleosomes was fuzzier in old cells, meaning that they tended to move slightly to the left or right along the DNA sequence. This indicates that the lack of steric hindrance from adjacent nucleosomes enables the nucleosomes to have more freedom in the precise DNA sequence that they chose to form on (Fig. 2A). About 3% of nucleosomes did move to completely new DNA positions during aging, moving specifically to DNA sequences that had a higher predicted ability to wrap around the histone octamer forming more energetically favorable nucleosomes (Fig. 2B). We were also interested in the functional consequences of such a profound loss of nucleosomes from the genome.

**Figure 2:**
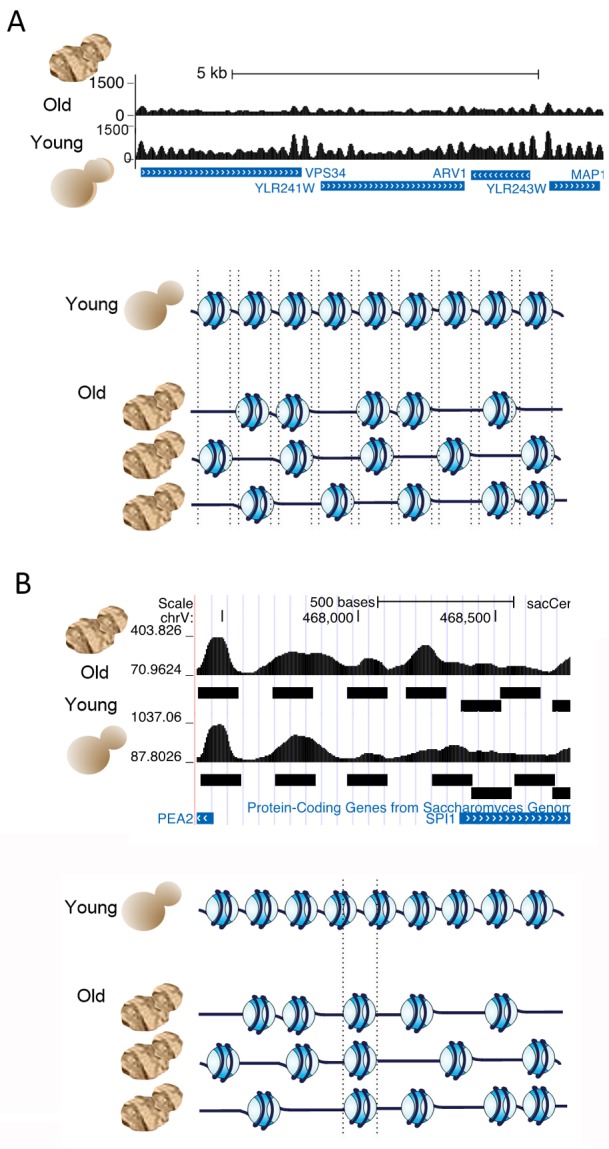
FIGURE 2: Changes in chromatin structure during aging. **(A)** Nucleosome occupancy is reduced by half over the entire yeast genome in old cells, while positioning of nucleosomes becomes fuzzier in old cells. The schematic at the bottom indicates that most nucleosome positions are retained within the population of old cells, while each cell has only half of the number of nucleosomes. The dotted lines indicate that the nucleosomes in old cells can move to the left or right of the positions in young cells in the absence of steric hindrance from adjacent nucleosomes. **(B)** Some nucleosomes move significantly during aging, as shown by the nucleosome positioning tracks above and the schematic below.

Earlier studies from our lab using model yeast genes had shown that loss of nucleosomes from promoter regions is sufficient to allow access to the transcriptional machinery, leading to transcriptional induction even in the absence of the sequence-specific transcriptional activators. Therefore we predicted that loss of half of the nucleosomes in old yeast should be accompanied by global transcriptional induction. However, this prediction is quite different from what previous analyses of transcriptional regulation during aging in yeast had shown, albeit in the absence of appropriate normalization controls. By spike-in controlled RNA-sequencing analysis, we found that every gene in the entire yeast genome was induced during aging. The more repressed that any gene was in young cells, the higher its fold induction of transcription during aging. This is not surprising given that it is the presence of histones that represses transcription, and loss of histones during aging would therefore relieve the transcriptional repression. For example, genes proximal to the telomeres are normally repressed by chromatin-mediated telomeric-silencing, and we found telomere-proximal genes to be among those most induced during aging. The global upregulation of all gene expression upon halving nucleosome occupancy on the genome highlights the important role that the chromatin structure plays in suppressing transcription, in order to enable subsequent transcriptional induction at the right time and place.

It has long been speculated that DNA damage plays a causal role in the aging process. Given the difficulty in isolation of useful numbers of old budding yeast, this link had largely been limited to a correlation between increased amounts of reactive oxygen species and age in yeast. We revisited the relationship between DNA damage and age, given that we were using an approach that enabled isolation of larger quantities of old yeast. Almost half of the old yeast had detectable amounts of the gamma-H2A marker of DNA damage, and approximately half of the old yeast had detectable amounts of DNA breaks. Whether there is a DNA repair defect in old cells or whether there is more DNA damage, or a combination of both, is unknown. Mapping of the gamma-H2A enrichment during aging found it to be enriched at two genomic locations, the first being at the ribosomal DNA (rDNA) locus. We also found a 15% increase in the gDNA content of the chromosomal region distal to the rDNA locus in old cells, indicating that potentially 15% of the old cells had an amplification that was caused by a DNA break within the rDNA locus. Furthermore, we mapped 20,000 translocations and found that translocations involving the rDNA locus were significantly increased during aging. These results reveal that DNA breaks within the rDNA are repaired inaccurately in old cells causing gross chromosomal rearrangements, which could potentially contribute to the aging process.

The second location that had indications of elevated levels of DNA damage during aging was much more unexpected, and was the mitochondrial DNA (mtDNA). The mtDNA was enriched for gamma-H2A during aging, which in itself was surprising given that H2A is a histone, and DNA within the mitochondria is not packaged with histones into nucleosomes. However, we were also able to map nucleosomes onto the mitochondrial genome during our nucleosome positioning analysis, indicating that at least some of the mtDNA is packaged into nucleosomes. The explanation for the chromatin packaging of the mtDNA came from our translocation mapping, which found that those translocations in old cells that do not involve the rDNA, involved the mtDNA. Ultimately, we used a functional assay to indicate that the rate of transfer of mtDNA into the nuclear genome is at least 100 fold increased during aging. It would appear that mtDNA transfers into the nuclear genome, presumably into sites of existing DNA breaks. Insertion of mtDNA into the genome would itself be mutagenic and may be deleterious. Another type of mutagenic DNA insertion event that we found to increase significantly was retrotransposition. This was a direct consequence of the induction of retrotransposon transcription that was caused by the histone loss. The retrotransposon-encoded RNAs are reverse transcribed into retrotransposon DNA genomes, which we found to be reinserted into non-typical insertion sites within the aging genome, doubling the overall retrotransposon content of old genomes.

It will be interesting to further investigate whether rDNA instability, retrotransposition and mitochondrial DNA insertion into the nuclear genome also increase during the aging of metazoans, and if so, whether this is related to alterations of chromatin structure, and whether it drives the aging process in eukaryotes. We speculate that the novel types of genome instability that we have uncovered, triggered by histone loss, will result in irreversible activation of the DNA damage checkpoint, causing cells to cease further cell divisions, leading to senescence.

